# Imprinting alterations in sperm may not significantly influence ART outcomes and imprinting patterns in the cord blood of offspring

**DOI:** 10.1371/journal.pone.0187869

**Published:** 2017-11-14

**Authors:** Li Tang, Zichao Liu, Ruopeng Zhang, Cunmei Su, Wenjuan Yang, Youlin Yao, Shuhua Zhao

**Affiliations:** 1 Department of Reproduction and Genetics, the First Affiliated Hospital of Kunming Medical University, Kunming, Yunnan Province, China; 2 Key Laboratory of Special Biological Resource Development and Utilization of Universities in Yunnan Province, Department of Life Science and Technology, Kunming University, Kunming, Yunnan Province, China; 3 Department of Reproductive Medicine, the First Affiliated Hospital of Dali University, Dali, Yunnan Province, China; 4 Yunnan Population and Family Planning Research Institute, Kunming, China; Peking University Third Hospital, CHINA

## Abstract

An increase in imprinting disorders in children conceived though assisted reproductive technologies (ARTs) has been the subject of several reports. The transmission of imprinting errors from the sperm of infertile fathers is believed to be a possible reason for the increased occurrence of these disorders. However, whether the imprinting alterations in sperm affect ART outcomes and the imprinting of offspring is unclear. In the current study, we analyzed the methylation of *H19*, *SNRPN* and *KCNQ1OT1* by pyrosequencing sperm samples from 97 infertile patients and 31 proven fertile males as well as cord blood samples from 13 infantswho were conceived by infertile parents through intracytoplasmic sperm injection (ICSI) and 30 healthy newborns who were conceived naturally. After four cases were excluded owing to the lack of a sequencing signal, the infertile patients were subgrouped into normal (69 cases) and abnormal (24 cases) imprinting groups according to the reference range set by the control group. Between the groups, there were no significant differences in ART outcomes. Significantly different levels of methylation were detected in *H19*, but none of the imprinted genes were determined to be outside of the methylation reference range set by the values derived from the naturally conceived controls. Three CpG loci were found to be significantly hypomethylated in the maternally imprinted gene *KCNQ1OT1* in two patients from the abnormal imprinting group, none of which were caused by sperm imprinting errors. In addition, the paternal *H19* gene exhibited discrepant methylation patterns between the sperm controls and the cord blood controls. Our data suggest that increased imprinting errors in the sperm of infertile patients do not have an obvious influence on ART outcomes or the imprinting of offspring.

## Introduction

It is estimated that more than 5 million infants resulting from assisted reproductive technologies (ARTs) have been born worldwide since 1978 when the first baby conceived via ART was born[1].However, the potential risks of ARTs are concerned for the effects of a genetic background of infertility as well as artificial operations performed at gametogenesis and in the early embryonic stages during which major epigenetic events occur[2].Adverse outcomes, including low birth weight and small size for gestational age, have been reported to occur at a greater frequency in infants conceived via in vitro fertilization (IVF) or intracytoplasmic sperm injection (ICSI)[3–6].Furthermore, positive links between ARTs and imprinting disorders have been verified with strong evidence[7–10].

Imprinting controls the allele-specific expression of genes based on parental origin by allele-specific methylation. Imprinted genes play important roles during fetal and placental growth, and tissue differentiation [11–14]. Aberrant imprinting leads to developmental disorders, including Beckwith-Wiedemann syndrome (BWS: OMIN130650), Angelman syndrome (AS: OMIN 105830) and Silver-Russell syndrome (SRS: OMIN 180860), which are associated with imprinted genes including *H19/IGF2*, *SNRPN* and *KCNQ1OT1*[15–17].

In mice, aberrant imprinting is observed in embryos after superovulation and the culture of embryos in different commercial media systems[18]. In humans, however, controversial results have been reported when comparing the imprinting in children conceived by IVF/ICSI with that in children conceived naturally. A meta-analysis of 18 studies has shown that there is an increase in imprinting disorders in children conceived though IVF and ICSI, but insufficient evidence has been reported to verify an association between ARTs and methylation in imprinted genes[10].By contrast, accumulating experimental evidence has linked aberrant imprinting in sperm to male infertility[17,19–27].Imprinting is typically reset at germ cell stages, during which the methylation of imprinted genes is removed and re-established. The new, sex-specific imprinting patterns are typically maintained during early embryonic development when genome-wide demethylation and de novo methylation occur[18].The methylation errors in the sperm of infertile men may be transmitted to offspring through ARTs, which could be another possible explanation for the increase in imprinting disorders observed in ART children. However, whether the imprinting errors in sperm affect ART outcomes and to what extent these errors are transmitted to offspring are unclear.

In the current study, we have analyzed the association between the methylation of imprinted genes, including *H19*, *SNPRN* and *KCNQ1OT1*, in sperm and ART outcomes in couples with male infertility. We have also investigated the relationship between the methylation patterns of imprinted genes in sperm and those in cord blood.

## Materials and methods

### Patients and sample collection

This study was approved by the Ethics Committee at the Yunnan Population and Family Planning Research Institute and performed in compliance with the Helsinki Declaration (2008). Written informed consent was obtained from the participants. For cord blood samples, written informed consent was obtained from parents.

Ninety-seven sperm samples were collected from patients diagnosed with male infertility who were undergoing ICSI treatment at the Yunnan Population and Family Planning Research Institute and the First Affiliated Hospital of Dali University from August 2014 to December 2016, including 38 severe oligozoospermic, 29 medium or mild oligozoospermic and 30asthenozoospermic patients. The diagnosis was based on the criteria established by the World Health Organization (WHO)(2010). The age range of the infertile male group was between 22 and 40 years. Patients with known genetic causes for infertility were excluded. Thirty-one semen samples from men who were proven fertile with normal semen parameters were collected as a control group. The control subjects were aged between 25 and 35 years.

Semen parameters were measured according to the 2010 WHO guidelines. Spermatozoa were purified by SpermGrad 45/90 (Vitrolife, Sweden) according to the manufacturer’s instructions.

Information regarding the ICSI-embryo transfer (ICSI-ET) treatment was collected, including the fertilization, good embryo, and clinical pregnancy rates. Telephone follow up was used to obtain information regarding the delivery, including the weeks of gestationjavascript:void(0);, birth weight and the presence/absence of birth defects.

Thirteen samples of cord blood were collected from infants born after ET. To establish a reference threshold for the normal methylation status, 30 cord blood samples were collected as controls from naturally conceived healthy newborns with birth weights between 2500 and 3800g following a normal full-term delivery.

### DNA isolation

Sperm were digested following a previously reported protocol[28].Sperm DNA and cord blood DNA were isolated using the QIAamp DNA Investigator Kit(Qiagen, Germany)and the DNeasy Blood & Tissue Kit (Qiagen, Germany), respectively. The DNA concentration was quantified with a NanoDrop 2000c. Genomic DNA was bisulfite-converted using the EpiTect Bisulfite Kit (Qiagen, Germany) according to the manufacturer’s instructions.

### Pyrosequencing

The PCR program consisted of a denaturing step of 3 min at 95°C followed by 45 cycles of 15 s at 95°C, 20 s at 54°Cand 30 s at 72°C, with a final extension of 5 min at 72°C.

The pyrosequencing samples were prepared using the Vacuum Prep workstation (Biotage AB, Uppsala, Sweden). The biotinylated amplicons were immobilized onto streptavidin Sepharose beads in the following reaction system:40 μl of the amplicon, 3 μl of streptavidin Sepharose HP beads (GE Healthcare), 37 μl of binding buffer [10 mM Tris-HCl, 2 M NaCl, 1 mM EDTA, 0.1% Tween-20, and Milli-Q (18.2 MΩ x cm) water,pH 7.6] and 15 μl of Milli-Q water. After one denaturation step and two washing steps using the Vacuum Prep workstation, the amplicons were transferred to a plate containing 0.4 μM sequencing primer in 40 μl of annealing buffer (20 mmol/l Tris-acetate and 2 mM magnesium acetate, pH 7.6). Pyrosequencing was performed using the PyroMark Gold Q96 Reagent and the PyroMark ID system (Qiagen, Germany), and sequences were analyzed with the Pyro Q-CpG™ software v. 1.0.9.PCR and sequencing primers were synthesized based on previously reported sequences[29,30].

### Statistical analyses

A one-way ANOVA was performed to assess the statistical significance of differences among groups in clinical parameters, including male age, female age, the number of eggs retrieved, fertilization rate, good embryo rate, sperm volume, sperm concentration, birth weight and delivery week. The methylation levels of imprinted genes or loci were also assessed using a one-way ANOVA. Tukey’sHSD post hoc correction was used to test pair-wise significant differences. In cases in which the homogeneity of variance showed significant differences, the Dunnett T3 test was applied. Clinical outcomes, including the clinical pregnancy, delivery and low birth weight rates, were analyzed using the chi-square test.A value of *P*<0.05 was considered to be significant. All statistical calculations were performed using IBM SPSS 19.

## Results

### Methylation of imprinted genes in sperm

The DNA methylation of one paternally (*H19*) and two maternally (*SNRPN* and *KCNQ1OT1*) methylated imprinted genes in sperm samples from 97 infertile males and 31 control subjects was analyzed by bisulfite pyrosequencing. Four CpGs in the differentially methylated region (DMR) of *H19*, five in the DMR of *SNRPN*, and six in the DMR of *KCNQ1OT1*were evaluated by pyrosequencing. The CpG data from four cases with severe oligozoospermia were not detected because of a low quantity of DNA. Among the imprinted genes, there were significant differences in the methylation levels of *H19* between the infertile males and the fertile controls (Table 1).

**Table 1:** Methylation level of studied genes in sperm.

Gene	infertile males, % (n = 94)	Controls,% (n = 30)
*H19*	91.6 ± 3.0*	93.1 ± 2.1
*SNRPN*	2.2 ± 5.0	1.3 ± 1.7
*KCNQ1OT1*	0.9 ± 1.7	0.6 ± 0.9

*P<0.05

### ART outcomes

A range of reference methylation of analyzed DMRs was set using the value of mean ± 2 standard deviation (SD) in control (S1 Table). The reference ranges of *H19*, *SNRPN* and *KCNQ1OT1* were 88.8–97.4%, 0–4.3% and 0–2.3%, respectively. Compared to these thresholds, 24 patients were considered to have imprinting errors: 15 had imprinting errors on *H19*;7had errors on *SNRPN*, and 7had errors on *KCNQ1OT1*. To investigate whether ART outcomes were associated with sperm imprinting, the infertile males were subgrouped into normal (n = 69) and abnormal (n = 24) imprinting groups according to the thresholds mentioned above. The abnormal imprinting group included7 severe oligozoospermic, 7 medium or mild oligozoospermic, and 10 asthenozoospermic patients. The outcomes of 112 patients undergoing IVF treatment at the same time were collected as controls. The ages of the males and females were not significantly different amongthe groups (Table 2). As expected, the sperm concentration was significantly lower in the normal imprinting and abnormal imprinting groups (*P*<0.001) than that in the fertile controls (Table 2).No significant differences were observed in the rates of fertilization, good embryo, clinical pregnancy, or delivery as well as birth parameters, including birth weight and delivery week, in both fresh and frozen ET cycles (Table 2).

**Table 2:** Clinical information and outcomes of IVF/ICSI-ET.

	IVF	Normal imprinting^a^	Abnormal imprinting^b^
Total (n)	112	69	24
Male age (year, mean ± SD)	34.7±5.1	34.1±5.9	36.5±6.2
Female age (year, mean ± SD)	32.7±4.7	30.9±4.8	33.8±5.4
Eggs (n, mean ± SD)	8.7±5.3	8.4±4.3	8.5±5.8
Fertilization rate (%, mean ± SD)	77.7±35.7	65.8±26.8	74.8±20.9
Good embryo rate (%, mean ± SD)	59.2±32.8	57.3±35.2	52.2±37.1
Semen volume(mL, mean ± SD)	3.4±1.7	3.4±1.6	3.2±1.5
Sperm concentration (x10^6^/mL, mean ± SD)	69.0±23.9	13.0±12.7***	16.0±19.5***
Fresh embryo transfer			
Cycle(n)	85	63	19
Clinical pregnancy rate (%)	40.0	34.9	36.8
Delivery rate (%)	34.1	31.7	31.6
Newborns (n)	38	25	8
Birth weight (Kg, mean ± SD)	2.84±0.61	2.76±0.73	2.93±0.56
Delivery week (week, mean ± SD)	38.0±2.7	37.5±3.3	38.8±1.2
Low birth rate (%)	18.4	32	25
Freezing embryo transfer			
Cycle of(n)	57	38	15
Clinical pregnancy rate (%)	43.8	42.1	46.7
Delivery rate (%)	33.3	34.2	33.3
Newborns (n)	25	17	6
Birth weight (Kg, mean ± SD)	3.05±0.54	2.84±0.49	2.98±0.40
Delivery week (week, mean ± SD)	38.5±1.6	37.8±1.2	38.8±1.1
Low birth rate (%)	16.0	17.6	0

^a^ infants were given birth by patients with normal imprinting in sperm.

^b^ infants were given birth by patients with abnormal imprinting in sperm.

***P< 0.001 in comparison to the IVF group.

### Methylation of imprinted genes in cord blood

As a control, the methylation of imprinted genes in 30 cord blood samples from infants who were conceived naturally was determined by sequencing (S2 Table). For *SNRPN*, *KCNQ1OT1*and *H19*,the mean methylation levels were found to be 42.5%±1.6%, 39.0%± 2.4%, and 53.9%±2.9%, respectively. The methylation level for the paternally methylated gene H19 was significantly higher than that for the maternally methylated genes *SNRPN* (*P*<0.001) and *KCNQ1OT1* (*P*<0.001).

**Fig 1:**
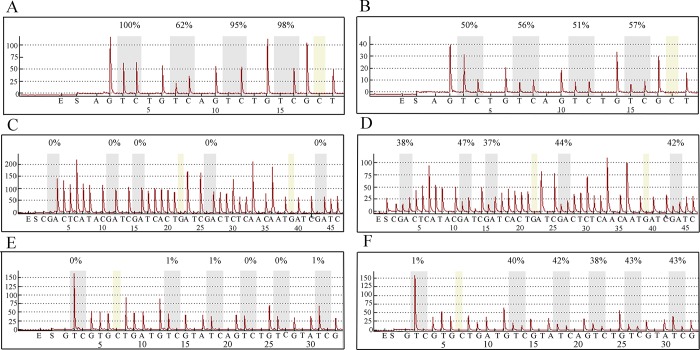
Pyrosequencing results of *H19* (A,B), *SNRPN* (C,D) and *KCNQ1OT1* (E,F)in sperm (A,C,E) and in corresponding cord blood samples (B,D,F) from case 86.

We compared the methylation of imprinted genes between infants who were conceived naturally and those conceived through ICSI (Table 3).No significant differences were found for *SNRPN*, *KCNQ1OT1*or *H19*. For *SNRPN* and *KCNQ1OT1*, no significant differences were found in the cord blood of offspring from the normal and abnormal imprinting groups. For *H19*, the methylation level was significantly lower in the abnormal imprinting group than that in the normal imprinting group (*P*<0.05), but no significant difference was found compared with the control group. Regarding the mean methylation levels of the imprinted genes, we did not find any case that was outside of the reference methylation range(the value of the mean±2 SD).However, for single CpG locus, we found that three loci of *KCNQ1OT1*intwo cases(case64 and case86) were extremely outside of the reference range. None of these errors were detected in the corresponding sperm samples (Table 4 and Fig 1).

**Table 3:** Methylation of studied genes in cord blood of offspring.

Gene	Infants conceived naturally,% (n = 30)	Infants conceived through ICSI,%		
		Normal imprinting ^a^ (n = 6)	Abnormal imprinting ^b^ (n = 7)	Total (n = 13)
*H19*	53.9 ± 2.9	55.5 ± 1.5	53.3 ± 1.2*	54.3 ± 1.7
*SNRPN*	42.5 ± 1.6	43.2 ± 1.2	42.5 ± 1.5	42.8 ± 1.4
*KCNQ1OT1*	39.0 ± 2.4	38.0 ± 2.1	38.0 ± 3.1	38.0 ± 2.6

^a^ infants were given birth by patients with normal imprinting in sperm.

^b^ infants were given birth by patients with abnormal imprinting in sperm.

*P< 0.05 in comparison to the normal imprinting group.

**Table 4:** Comparison of methylation of CpGs in matching sperm and cord blood samples.

case	sample	*H19*					*SNRPN*						*KCNQ1OT1*						
		CpG1	CpG2	CpG3	CpG4	mean	CpG1	CpG2	CpG3	CpG4	CpG5	mean	CpG1	CpG2	CpG3	CpG4	CpG5	CpG6	mean
4	sperm	100	72	96	96	91.0	**10**	**12**	**10**	**11**	**10**	**10.6**	1	1	1	2	1	1	1.2
	cord blood	48	52	55	52	51.8	44	43	41	42	39	41.8	38	41	42	43	41	42	41.2
32	sperm	**83**	100	100	100	95.8	**11**	**10**	**21**	**21**	**22**	**17.0**	0	0	0	0	0	0	0
	cord blood	53	54	57	55	54.8	44	45	41	42	41	42.6	36	36	40	36	38	40	37.7
47^b^	sperm	100	83	100	**70**	**88.3**	**20**	**21**	**20**	**19**	**19**	**19.8**	0	0	**3**	0	0	0	0.5
	cord blood	54	53	52	56	53.8	46	47	43	45	44	45.0	40	42	45	44	40	42	42.2
	cord blood	51	57	53	55	54.0	43	44	42	43	41	42.6	38	35	39	37	38	40	37.8
64	sperm	100	74	97	97	92.0	4	4	3	4	4	3.8	**3**	3	**3**	**4**	3	0	**2.7**
	cord blood	53	55	52	54	53.5	44	45	43	45	40	43.4	37	38	**26**^**a**^	38	38	**25** ^**a**^	33.7
71	sperm	100	75	98	97	92.5	0	0	0	0	0	0	**4**	**5**	**4**	**9**	**4**	0	**4.3**
	cord blood	50	54	49	53	51.5	41	42	40	40	38	40.2	38	37	41	39	38	40	38.8
86	sperm	100	**62**	**95**	98	**88.7**	0	0	0	0	0	0	0	1	1	0	0	1	0.5
	cord blood	50	56	51	57	53.5	38	47	37	44	42	41.6	**1** ^**a**^	40	42	38	43	43	34.5

Bold text highlights the CpG sites or mean levels where methylation is abnormal in sperm or in cord blood.

^a^ the CpG sites at which methylation is abnormal in cord blood.

^b^ cord blood samples were collected from twins born after ICSI-ET.

### Association of methylation in sperm and cord blood samples

**Fig 2:**
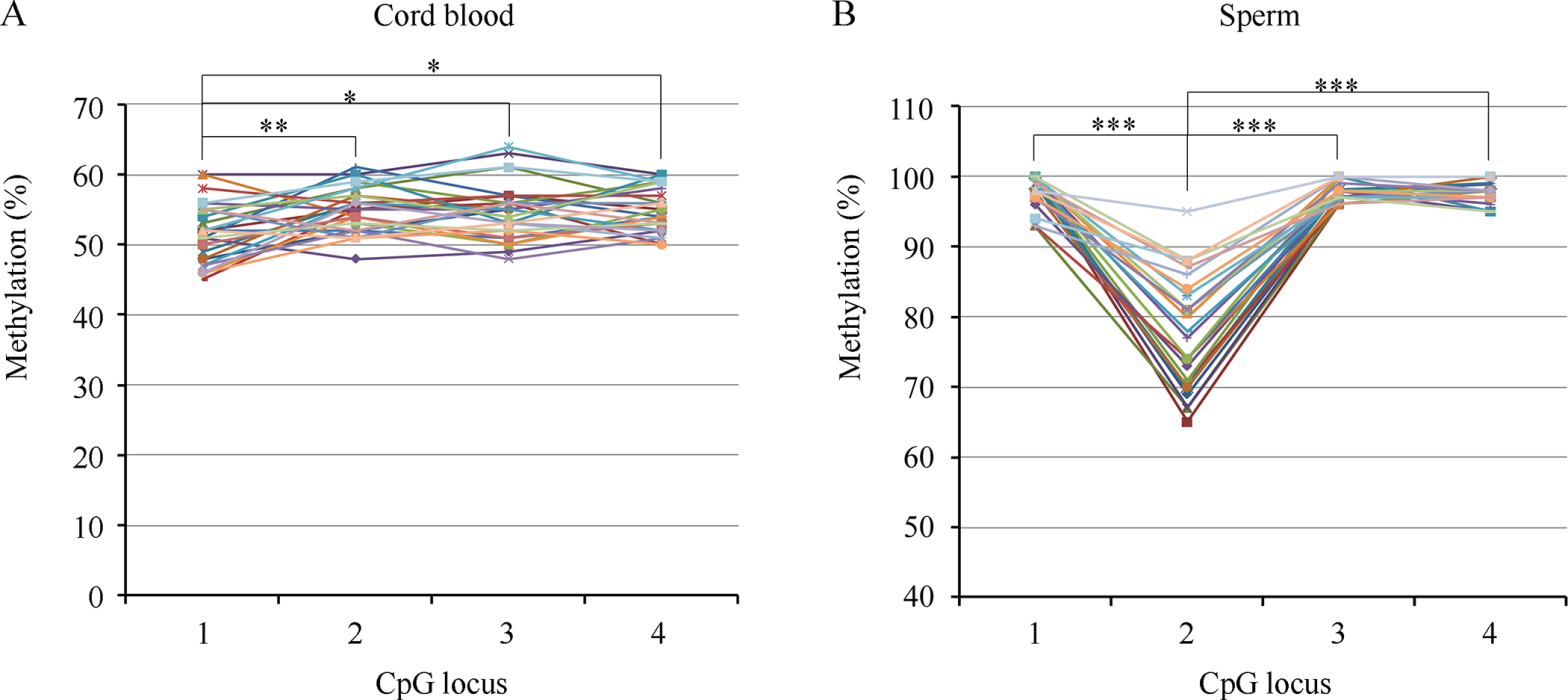
**Methylation pattern at the *H19* DMR in cord blood from natural conceived infants (A) and in sperm from proven fertile males (B).** A mean level of methylation for each CpG position was tested by pryosequencing. Circles represent the CpG positions and each line represents an individual sample analysis. (A) the mean methylation level of CpG1 is significantly low compared with CpG2 (**P< 0.01), CpG2 (*P< 0.05) or CpG3 (*P< 0.05). (B) the mean methylation level of CpG2 is significantly low compared with the other CpG positions (***P< 0.01).

As shown in Fig 2, the methylation patterns of H19 were inconsistent between the sperm and cord blood samples. In the paternal sperm, the lowest and most diverse methylation values were detected in CpG2, which was significantly different compared with the other three loci. In the cord blood of offspring, however, the methylation level of CpG2 was high and uniform. By contrast, the methylation level of CpG1 was significantly lower than that of the other loci.

To further investigate the relationship between imprinting in the sperm and imprinting in the cord blood of offspring, we performed a correlation analysis for each CpG site between matching sperm and cord blood samples. Significant correlations were not found.

## Discussion

The available epidemiological and experimental data regarding the association between aberrant methylation of imprinted genes in males and infertility, including alterations of sperm concentration, motility, and morphology, have raised the concern that these imprinting errors may be transmitted to offspring and lead to imprinting disorders, including BWS, SRS, and AS[17,19–27]. In the current study, we sought to address the possibility of transmitting imprinting errors in sperm to offspring. Our main findings were as follows: 1) the aberrant methylation of H19was associated with oligozoospermia (Table 1); 2) the outcomes did not show significant differences among the IVF-ET control, normal imprinted sperm and abnormal imprinted sperm groups (Table 2); 3) the methylation of *H19* in cord blood was significantly lower in the abnormal imprinting group than that in the normal imprinting group but was not significantly different from controls who were conceived normally(Table 3); and 4) no correlation was found between the imprinting patterns in sperm and those in cord blood (Table 4 and Fig 2).

Recent studies have suggested that there is an increased incidence of typically rare imprinting disorders, including BWS, SRS and AS, in children conceived through ARTs[7–10].The artificial treatments involved in ARTs at the time when major epigenetic events occur are believed to be responsible for the increase in imprinting disorders. Alternatively, increased imprinting aberrations in the sperm of infertile males have been reported previously and in the current study[17,19–26]. These aberrations have a good chance of being passed on to offspring because such imprinted methylation is maintained during the preimplantation stage of development when DNA methylation erasure occurs[18]. Indeed, Kobayashi et al. have found identical alterations in aborted conceptus samples and the corresponding parental sperm samples and concluded that the abnormal methylation of imprinted genes in offspring could be inherited directly from the father[27]. In addition, Kagami et al. have reported that in a patient with SRS, four out of the eight abnormally methylated cytosines are also detected in the father[31]. In the current study, we found that the methylation level of *H19*was significantly decreased in infants whose fathers produced sperm with imprinting errors (Table 3), suggesting an influence of paternal sperm methylation status on the imprinting of offspring. However, additional evidences indicated that the effect was limited. First, although significant differences in *H19*methylation levels in the cord blood were found between the offspring of normal and abnormal imprinting groups, no significant difference was found when compared with naturally conceived controls (Table 3). In addition, no case was determined to be out of range compared to the reference range established by the controls. Second, the methylation pattern of *H19* in cord blood was different from that in sperm (Fig 2). In sperm, CpG2was significantly less methylated than the other 3 loci. In cord blood,CpG2 was highly methylated, while CpG1 exhibited a significantly lower methylation level. Third, none of the imprinting errors in the cord blood of offspring could be traced back to the father’s sperm (Table 4). Only three CpGs of *KCNQ1OT1* in two infants were found to be hypomethylated. It is impossible for infants to inherit these errors from fathers because *KCNQ1OT1*is a maternally imprinted gene. There is other evidence against a significant influence of paternal sperm imprinting on offspring. Significantly higher frequencies of imprinting errors were not found in babies conceived via IVF or even ICSI, which is typically used in cases involving male infertility, compared with babies conceived through natural means[32–36].Furthermore, low frequencies of imprinting defects have been detected even in ICSI children who were born small for their gestational age[37].These findings suggest that imprinting defects are not as frequently observed in children conceived via ARTs, as is observed in sperm. Therefore, we conclude that the effect of paternal sperm imprinting on the imprinting of offspring is limited, although the possibility of directly transmitting imprinting errors has not been excluded.

It is likely that the imprinting errors in sperm are selectively discarded or corrected during development. Epigenetic mosaicism in the sperm of oligoasthenozoospermic men has been reported by Laurentino and colleagues[20]. In addition, more imprinting errors have been detected in sperm samples that do not result in a pregnancy than in samples that lead to a live birth[38].One explanation for these findings is that spermatozoa with imprinting errors have less fertilization ability compared with normal spermatozoa and are selectively discarded during fertilization. However, the natural selection mechanism is bypassed when ICSI, the primary choice for oligoasthenozoospermic men, is used for fertilization. It has been reported that the epimutation is not decreased even in spermatozoa that have been selected for intracytoplasmic morphologically selected sperm injection (IMSI), which uses a magnification that is approximately 15 times higher than that used in standard ICSI[38]. In the current study, the imprinting status was found to be normal in seven cord blood samples from infants conceived via ICSI whose fathers were diagnosed with oligozoospermia and had imprinting errors in their sperm (Table 4). Even so, the possibility of fertilization by a spermatozoon with normal methylation pattern is high, as none of methylation alteration is presented in totality spermatozoa studied. Thus, in current study, we cannot discard the possibility that all infants are from embryos fertilized by normal spermatozoon because of the limited sample size. But when comparing the methylation patterns between samples of sperm and cord blood, we have found distinct patterns at *H19*. The methylation level of CpG2 is significantly lower than that of the other loci in sperm. If the methylation status of sperm directly transmits to the offspring, the methylation level of corresponding locus in cord blood would show some remains, which are not found in the current study (Fig 2). Thus, epigenetic mosaicism is probably not the only reason for decreases in imprinting errors. After fertilization, it has been reported that the frequency of imprinting errors is high both in embryos that failed to develop and in aborted conceptus samples after ARTs in humans[27,39]; additionally, in mice, imprinting defects are frequently observed at the blastocyst stage but not during mid gestation or in placentas derived from superovulated mothers [40].These findings suggest the alternative explanation that embryos or cells that contain imprinting aberrations are eliminated during development, resulting in normal imprinting patterns in the surviving offspring. Nevertheless, imprinting remodeling is not excluded, although the imprinting status is maintained during the genome-wide demethylation and de novo methylation that occurs after fertilization, as the methylation levels of imprinted genes are varied in different tissues[30].Sanchez-Delgado and colleagues have demonstrated in primates that sperm-derived methylation is reprogrammed by the blastocyst stage, while oocyte-derived differences in methylation persist[41].It is worth noting that tissue specific methylation is possible for the different imprinting patterns observed in cord blood samples. Tabano et al have observed that the methylation levels of *H19* DMRs is different between embryonic (cord blood) and extraembryonic (placenta and umbilical cord) tissues [42]. Thus, samples from different tissues are necessary for further study. In conclusion, how and to what extent imprinting errors in sperm influence ART-derived offspring are far from being elucidated.

The current study is limited by its sample size. A large-scale cohort study is required to determine the probability of transmitting imprinting errors in sperm to offspring and the effect of these errors on ART outcomes and the health of the offspring. In addition, the methylation status of imprinted genes was detected by bisulfite pyrosequencing, which quantitatively determined the average methylation levels at each CpG site but could not distinguish the methylation levels that are specific to the paternal and maternal alleles. For this reason, imprinting errors will be hidden if the genes are hypomethylated on one allele and hypermethylated on the other, which has been reported in preimplantation embryos[39].Thus, new methods are required for further study. For example, deep bisulfite sequencing not only detects methylation at the level of a single molecule but also distinguishes the two parental alleles by a single nucleotide polymorphism (SNP) in the DMR[20].

In summary, it appears thatimprinting errors are frequently observed in the sperm of males undergoing ARTs, especially ICSI treatment, but the transmission ofthese errors to offspringis not frequently observed, at least not at the loci of *H19*,*SNPRN*, and *KCNQ1OT1*, which were investigated in this study. Our data and that of previous reports support the need for larger cohort studies to determine the modes of transmission and consequences of sperm imprinting errors in ART patients.

## Supporting information

S1 Table Methylation levels for imprinted genes in sperm from fertile controls. (PDF)

S2 Table Methylation levels for imprinted genes in cord blood from infants conceived naturally. (PDF)
