# Supplementary material for: Imprinting alterations in sperm may not significantly influence ART outcomes and imprinting patterns in the cord blood of offspring
Source: PLoS One. 2017 Nov 14;12(11):e0187869. doi: 10.1371/journal.pone.0187869 (PMC5685618; doi:10.1371/journal.pone.0187869)
Supplement: S1 Table — (PDF) [file pone.0187869.s001.pdf]

S1 Table Methylation levels for imprinted genes in sperm from fertile controls.

ID	KCNQ1OT1							SNRPN						H19				
	1	2	3	4	5	6	average	1	2	3	4	5	average	1	2	3	4	average
sc1	0	0	0	0	0	0	0.00	3	3	3	2	2	2.60	100	80	100	100	95.00
sc2	0	3	0	2	0	0	0.83	2	0	0	0	0	0.40	93	67	96	97	88.25
sc3	0	0	0	3	0	0	0.50	3	3	2	2	3	2.60	98	70	97	100	91.25
sc4	0	4	3	3	3	0	2.17	3	0	0	0	0	0.60	100	67	97	97	90.25
sc5	0	3	0	0	0	0	0.50	3	3	2	3	0	2.20	100	74	100	95	92.25
sc6	0	3	3	4	0	0	1.67	2	0	0	0	0	0.40	100	88	97	95	95.00
sc7	4	6	4	3	3	4	4.00	4	2	2	2	2	2.40	100	71	100	100	92.75
sc8	0	4	3	3	0	0	1.67	3	2	2	2	2	2.20	100	77	98	96	92.75
sc9	0	0	0	0	0	0	0.00	4	5	5	4	4	4.40	100	74	100	100	93.50
sc10	0	0	0	0	0	0	0.00	2	2	2	1	2	1.80	100	81	97	98	94.00
sc11	0	0	0	0	0	0	0.00	2	2	2	1	0	1.40	93	74	100	100	91.75
sc12	1	0	2	0	2	0	0.83	3	3	3	2	3	2.80	100	74	96	98	92.00
sc13	0	0	0	0	0	0	0.00	1	0	0	0	0	0.20	98	88	100	100	96.50
sc14	0	0	1	2	0	0	0.50	2	1	2	2	2	1.80	100	83	97	97	94.25
sc15	0	0	0	0	0	0	0.00	2	2	2	0	2	1.60	94	88	100	100	95.50
sc16	1	0	0	0	0	0	0.17	2	2	2	1	2	1.80	96	73	98	95	90.50
sc17	2	0	0	0	3	0	0.83	2	2	1	2	2	1.80	97	84	98	97	94.00
sc18	0	0	0	0	0	0	0.00	1	0	0	0	0	0.20	94	88	100	98	95.00
sc19	0	0	0	2	0	0	0.33	2	2	2	2	2	2.00	100	71	96	97	91.00
sc20	0	0	0	0	0	0	0.00	2	0	0	0	0	0.40	99	70	98	99	91.50
sc21	0	0	0	0	0	0	0.00	0	0	0	0	0	0.00	97	81	99	98	93.75
sc22	2	0	2	0	0	0	0.67	2	1	2	2	2	1.80	100	65	96	97	89.50

sc23	2	0	0	2	0	0	0.67	0	0	0	0	0	0.00	97	81	98	98	93.50
sc24	0	0	0	0	0	0	0.00	2	1	1	1	2	1.40	100	74	98	98	92.50
sc25	2	0	0	0	2	0	0.67	2	2	2	2	2	2.00	100	81	98	98	94.25
sc26	0	0	0	0	0	0	0.00	2	3	2	0	2	1.80	93	86	100	100	94.75
sc27	0	2	0	0	2	0	0.67	4	3	3	3	3	3.20	99	78	97	97	92.75
sc28	0	0	0	0	0	0	0.00	1	0	0	0	0	0.20	100	69	97	97	90.75
sc29	2	2	0	2	0	2	1.33	3	0	4	0	0	1.40	99	87	96	97	94.75
sc30	0	0	0	0	5	0	0.83	1	1	1	0	0	0.60	98	95	100	100	98.25
sc31	0	0	0	0	0	0	0.00	6	5	5	5	8	5.80	100	83	97	99	94.25
