# Supplementary material for: Imprinting alterations in sperm may not significantly influence ART outcomes and imprinting patterns in the cord blood of offspring
Source: PLoS One. 2017 Nov 14;12(11):e0187869. doi: 10.1371/journal.pone.0187869 (PMC5685618; doi:10.1371/journal.pone.0187869)
Supplement: S2 Table — (PDF) [file pone.0187869.s002.pdf]

**S2 Table Methylation levels for imprinted genes in cord blood from infants conceived naturally.**

| ID | KCNQ1OT1 | | | | | | | SNRPN | | | | | | H19 | | | | |
|---|---|---|---|---|---|---|---|---|---|---|---|---|---|---|---|---|---|---|
| | 1 | 2 | 3 | 4 | 5 | 6 | average | 1 | 2 | 3 | 4 | 5 | average | 1 | 2 | 3 | 4 | average |
| cc1 | 40 | 39 | 42 | 38 | 40 | 40 | 39.83 | 43 | 44 | 41 | 43 | 40 | 42.20 | 48 | 52 | 55 | 52 | 51.75 |
| cc2 | 37 | 39 | 41 | 38 | 39 | 43 | 39.50 | 44 | 43 | 43 | 42 | 41 | 42.60 | 52 | 55 | 57 | 55 | 54.75 |
| cc3 | 38 | 38 | 39 | 37 | 39 | 39 | 38.33 | 43 | 45 | 41 | 44 | 39 | 42.40 | 53 | 58 | 61 | 56 | 57.00 |
| cc4 | 34 | 36 | 37 | 37 | 37 | 38 | 36.50 | 45 | 44 | 41 | 42 | 42 | 42.80 | 60 | 60 | 63 | 60 | 60.75 |
| cc5 | 41 | 43 | 44 | 40 | 43 | 43 | 42.33 | 42 | 43 | 42 | 44 | 40 | 42.20 | 49 | 56 | 53 | 51 | 52.25 |
| cc6 | 38 | 37 | 40 | 37 | 40 | 41 | 38.83 | 44 | 44 | 44 | 43 | 40 | 43.00 | 48 | 57 | 55 | 52 | 53.00 |
| cc7 | 38 | 39 | 39 | 39 | 40 | 41 | 39.33 | 43 | 44 | 43 | 44 | 40 | 42.80 | 51 | 61 | 57 | 54 | 55.75 |
| cc8 | 34 | 32 | 36 | 31 | 34 | 36 | 33.83 | 39 | 40 | 39 | 38 | 38 | 38.80 | 45 | 55 | 56 | 50 | 51.50 |
| cc9 | 40 | 40 | 43 | 42 | 41 | 44 | 41.67 | 42 | 45 | 41 | 43 | 40 | 42.20 | 56 | 59 | 56 | 59 | 57.50 |
| cc10 | 35 | 37 | 40 | 39 | 38 | 37 | 37.67 | 40 | 38 | 36 | 39 | 34 | 37.40 | 51 | 48 | 49 | 52 | 50.00 |
| cc11 | 39 | 39 | 43 | 39 | 41 | 44 | 40.83 | 43 | 44 | 44 | 44 | 40 | 43.00 | 54 | 60 | 53 | 60 | 56.75 |
| cc12 | 33 | 35 | 37 | 38 | 35 | 35 | 35.50 | 44 | 44 | 44 | 44 | 41 | 43.40 | 60 | 54 | 50 | 54 | 54.50 |
| cc13 | 36 | 37 | 40 | 39 | 37 | 40 | 38.17 | 43 | 45 | 41 | 43 | 42 | 42.80 | 52 | 52 | 51 | 54 | 52.25 |
| cc14 | 38 | 37 | 40 | 43 | 40 | 40 | 39.67 | 44 | 44 | 42 | 42 | 41 | 42.60 | 58 | 56 | 57 | 57 | 57.00 |
| cc15 | 37 | 37 | 40 | 39 | 37 | 40 | 38.33 | 45 | 45 | 42 | 45 | 41 | 43.60 | 51 | 53 | 50 | 55 | 52.25 |
| cc16 | 39 | 40 | 40 | 38 | 40 | 40 | 39.50 | 43 | 43 | 39 | 42 | 38 | 41.00 | 56 | 55 | 55 | 58 | 56.00 |
| cc17 | 47 | 45 | 47 | 48 | 46 | 46 | 46.50 | 45 | 48 | 46 | 47 | 44 | 46.00 | 47 | 56 | 55 | 52 | 52.50 |
| cc18 | 35 | 37 | 39 | 35 | 38 | 41 | 37.50 | 44 | 43 | 43 | 43 | 40 | 42.60 | 46 | 54 | 50 | 54 | 51.00 |
| cc19 | 39 | 40 | 42 | 40 | 40 | 40 | 40.17 | 44 | 44 | 40 | 44 | 39 | 42.20 | 55 | 51 | 56 | 56 | 54.50 |
| cc20 | 36 | 37 | 40 | 41 | 38 | 41 | 38.83 | 42 | 45 | 40 | 42 | 39 | 41.60 | 50 | 54 | 51 | 53 | 52.00 |
| cc21 | 38 | 38 | 39 | 38 | 38 | 38 | 38.17 | 42 | 45 | 42 | 44 | 41 | 42.80 | 55 | 57 | 54 | 59 | 56.25 |
| cc22 | 40 | 39 | 42 | 42 | 41 | 42 | 41.00 | 43 | 44 | 44 | 43 | 39 | 42.60 | 47 | 52 | 48 | 51 | 49.50 |

| | | | | | | | | | | | | | | | | | |
|---|---|---|---|---|---|---|---|---|---|---|---|---|---|---|---|---|---|
| cc23 | 36 | 37 | 39 | 40 | 38 | 37 | 37.83 | 44 | 45 | 43 | 43 | 43 | 43.60 | 52 | 58 | 64 | 59 | 58.25 |
| cc24 | 34 | 36 | 38 | 35 | 35 | 37 | 35.83 | 48 | 49 | 44 | 45 | 44 | 46.00 | 46 | 51 | 52 | 50 | 49.75 |
| cc25 | 35 | 37 | 40 | 40 | 39 | 41 | 38.67 | 43 | 45 | 42 | 43 | 42 | 43.00 | 52 | 51 | 53 | 51 | 51.75 |
| cc26 | 38 | 39 | 42 | 38 | 41 | 41 | 39.83 | 43 | 44 | 40 | 42 | 39 | 41.60 | 55 | 52 | 56 | 53 | 54.00 |
| cc27 | 39 | 41 | 45 | 40 | 42 | 45 | 42.00 | 44 | 42 | 42 | 42 | 40 | 42.00 | 51 | 53 | 52 | 53 | 52.25 |
| cc28 | 34 | 36 | 39 | 38 | 38 | 36 | 36.83 | 44 | 46 | 44 | 44 | 43 | 44.20 | 46 | 56 | 53 | 52 | 51.75 |
| cc29 | 39 | 42 | 41 | 35 | 37 | 42 | 39.33 | 45 | 45 | 42 | 43 | 41 | 43.20 | 56 | 59 | 61 | 59 | 58.75 |
| cc30 | 35 | 37 | 39 | 38 | 41 | 38 | 38.00 | 42 | 43 | 44 | 42 | 39 | 42.00 | 52 | 51 | 53 | 56 | 53.00 |
